# State-of-the-art preclinical techniques to study the impact of spreading depolarizations in awake rodents

**DOI:** 10.1186/s10194-025-02121-0

**Published:** 2025-08-29

**Authors:** Alejandro Labastida-Ramirez, Neela K. Codadu, Kagan Agan, Robert C. Wykes

**Affiliations:** 1 Division of Neuroscience & Centre for Nanotechnology in Medicine, Faculty of Biology, Medicine, and Health, University of Manchester, Manchester, M13 9LT UK; 2 Geoffrey Jefferson Brain Research Centre, Manchester Academic Health Science Centre, Northern Care Alliance NHS Foundation Trust, University of Manchester, Manchester, UK; 3 Department of Clinical and Experimental Epilepsy, University College London, Queen Square Institute of Neurology, London, WC1N 3BG UK; 4 Experimental Animals Application and Research Center, Duzce University, 81620 Duzce, Turkey

**Keywords:** Anesthesia, Cortical spreading depolarization, DC-coupled electrophysiology, Functional ultrasound, Migraine, Optogenetics, Wireless telemetry

## Abstract

**Background:**

Understanding the mechanisms of pathological brain network activity and the efficacy of therapies requires testing hypothesis in vivo, where brain circuitry remains preserved. Therefore, animal models are a key tool in the study of primary neurological disorders such as migraine, stroke and epilepsy. These models not only have advanced our understanding of the underlying neurobiology of these disorders but have also provided novel pharmacological targets and insights on shared pathophysiological processes such as spreading depolarizations (SD). SD, the electrographic correlate of migraine with aura, are transient waves of near-complete neuroglial depolarization associated with transmembrane ionic and water shifts.

**Body:**

Many studies investigating the impact of SD in preclinical models have done so in the presence of anesthesia. However, the use of anesthesia is a well-known confounding factor that not only influences SD threshold or frequency but also SD-evoked hemodynamic responses as common anesthetics affect cerebral blood flow and neurovascular coupling, limiting translation. Therefore, here we discuss research methods that have recently been developed or refined to allow the study of SD in awake rodents with a focus on migraine with aura. We discuss advantages, limitations and also efforts made to transition towards minimally-invasive procedures. Methods include optogenetic approaches to induce SD, multisite high-fidelity DC-coupled electrophysiological recordings, and measurements of neurovascular signals detected at both mesoscopic/macroscopic (e.g., fluorescent reporters, functional ultrasound system) and microscopic levels (e.g., two-photon microscopy, miniscopes). Additionally, we discuss continuous wireless telemetry recordings to detect spontaneous SD frequency over weeks to months in freely moving animals.

**Conclusion:**

Implementation of these methods in awake brain will close the translational gap and improve the relevance of preclinical animal models.

## Migraine with aura and the role of spreading depolarizations

Epidemiological studies have consistently shown that in approximately one-third of migraine patients, headaches are preceded by an aura which denotes transient visual, sensory or other focal neurological symptoms that spread gradually, lasting 5–60 min [1, 2]. Spreading depolarization (SD), the pathophysiological mechanism underpinning migraine with aura, is a slow wave of abrupt, sustained neuroglial depolarization in grey matter of the central nervous system, followed by long-lasting suppression of neuronal activity [3], termed spreading depression. The propagation velocity of the SD wave in the human cerebral cortex is approximately 2–5 mm/min, which correlates with the observed spread of the migraine aura [4–7]. SDs self-propagate in the grey matter by means of contiguity, regardless of arterial territories or functional divisions [8], however, they seem to be spatially restricted along a single sulcus or gyri in gyrencephalic brains [9].

SDs are characterized by the collapse of ion homeostasis, profound disruption of transmembrane ionic gradients and the release of neurotransmitters, neuropeptides and other molecules from cellular compartments [10]. In migraine with aura these changes lead to suppression of spontaneous electrical activity in the region and brief dilation of pial arteries, resulting in two well-known clinical observations, spreading depression of electrocorticographic activity and temporal increase in cerebral blood flow (CBF), respectively [1, 7]. The underlying cause(s) of SD induction in migraine with aura is only partly understood. However, diverse genetic mutations in neurons and glial cells underlying familial hemiplegic migraine type 1–3 (*CACNA1A*,* ATP1A2* or *SCN1A*, encoding different subunits of channels such as P/Q-type calcium channel, Na/K pump and Na channel), result in cortical hyperexcitability and all present with spontaneous SDs and increased susceptibility to SDs [11–13].

Different research groups have shown in rodents that SDs also contribute to the late activation and sensitization of trigeminal nociceptive afferents innervating the cranial meninges, the mechanism underlying the onset of migraine headache [14–16]. SDs trigger meningeal neurogenic inflammation, a peripheral response comprised of increased capillary permeability leading to plasma protein extravasation, activation of resident immune cells and arterial vasodilation (reviewed in [17]). The mechanisms underlying the meningeal responses to SDs are also incompletely understood, but presumably involve the release of nociceptive molecules (e.g., glutamate, potassium) in the cortical parenchyma during the passage of the SD wave, and their diffusion or bulk flow into the subarachnoid space and subsequent action upon dural trigeminal afferents [14, 17]. Moreover, it has been recently shown that after SDs, subarachnoid cerebrospinal fluid carries nociceptive signals (i.e., calcitonin gene-related peptide, CGRP) from the cortex directly to the cell bodies in the trigeminal ganglia, activating nociceptors through a direct pathway that bypasses meningeal trigeminal afferents [15].

Animal models of migraine with aura have greatly contributed to our current understating of the neurobiological mechanisms of aura. Typically, preclinical rodent studies that investigate the impact of SD have done so under anesthesia [18, 19]. However, the use of anesthesia is a well-known confounding factor (see below) that not only influences SD properties but also SD-mediated hemodynamic responses as common anesthetics affect CBF and neurovascular coupling (the term used to describe alterations in CBF that occur in response to neuronal activity), limiting translation [19–21]. Additionally, performing experiments in awake head-fixed, tethered, or freely moving animals allows researchers to study SD under more naturalistic conditions. There is a critical need to develop minimally or non-invasive methods for inducing and detecting SD, both to reduce experimental artifacts and to better study SD-related pathophysiology. For example, while SD is known to trigger pro-inflammatory gene expression, previous studies have relied on invasive techniques that independently induce inflammation, confounding the specific contribution of SD [22–24].

In this review, we discuss state-of-the-art preclinical methods recently developed or refined to study SDs in awake (i.e., anesthesia-free) rodents, highlighting their advantages, limitations, and the ongoing efforts to minimize invasiveness, with additional considerations for applications in migraine research.

## Effects of anesthesia on CBF and SDs

Anesthesia is an established confounding factor in preclinical *in vivo* research, as common anesthetics have profound effects on the systemic (e.g., mean arterial pressure) and cerebral physiological parameters of animals, limiting translation [19–21]. For instance, inhalation anesthetics (e.g., isoflurane), one of the most used classes of anesthetics in rodent research, have been shown to either increase or inhibit CBF depending on the dose [19]. Similarly, common injectable anesthetics such as pentobarbital and propofol reduce dose-dependently CBF [19]. The effects of anesthesia on neurovascular coupling are also complex; however, the predominant response reported with most frequently used anesthetics is a decrease in amplitude of the hemodynamic responses [19].

Anesthetics can also affect SD properties such as SD waveform, frequency, propagation speed, duration and threshold (reviewed in [19, 25]). They influence SD susceptibility in a dose-dependent manner although variability has also been reported, for example, propofol, ketamine and inhalation anesthetics suppress SDs, whereas barbiturates and urethane do not seem to suppress SDs [19, 26]. Although anesthetics do not seem to have a direct effect on SDs-evoked hemodynamic responses, anesthesia can indirectly influence the hemodynamic response by changing systemic blood pressure and/or resting CBF [1, 25]. Thus, for the reasons outlined above, SD induction, along with the detection and monitoring of downstream effects, such as blood flow and inflammation, in awake rodents has gained momentum. This approach seeks to reduce the confounding effects of anesthesia and to improve both understanding and translational efficacy of therapies.

## SD induction techniques

Understanding the mechanisms of SD requires reliable induction and detection methods that minimally disrupt the brain’s natural environment. Non-invasive approaches are critical because traditional invasive techniques such as electrode insertion or chemical application can themselves alter cortical excitability, induce inflammatory responses, and confound the physiological dynamics of SD. By minimizing tissue damage and preserving normal neurovascular coupling, non-invasive strategies allow for more accurate modelling of spontaneous or disease-related SD events, such as those occurring in migraine, stroke, and traumatic brain injury. In rodents, the small size and susceptibility of brain structures to perturbation necessitate the development and application of non-invasive methodologies, which enhance translational relevance, improve experimental reproducibility, and enable longitudinal studies by allowing repeated assessments of the same animals over time.

Inducing SD has traditionally relied on methods like electrical stimulation or the application of chemicals to manipulate neural activity. While effective, these techniques often face limitations, including invasiveness, lack of specificity, and difficulty in controlling the precise location and timing of the induced depolarization (Table 1). Electrical stimulation can cause broad activation of neural networks, while chemical methods may result in off-target effects, leading to variable results [25].

**Table 1:** Comparison of common SD induction techniques

Method	Mechanism	Advantages	Limitations
Mechanical stimulation	Localized injury to neural tissue (e.g. needle pinprick)	Effective at inducing SDs; simple to apply	Injury confounds studies; inability to repeat SD waves
Electrical stimulation	Application of current to neural tissue	Effective at inducing SDs; relatively simple to apply	Broad, non-specific activation; invasive; variable results
Chemical application	Topical application of depolarizing agents (e.g., KCl)	Can reliably induce SD with simple tools	Off-target effects; diffusion limits spatial precision; invasive
Optogenetics	Light activation of neurons expressing light-sensitive ion channels (e.g., channelrhodopsins)	High spatial and temporal precision; cell-type specific targeting; adaptable to non-invasive delivery	Requires genetic modification; needs specialized equipment (LEDs, optics)

Optogenetics offers a powerful and precise method to induce SD by using light to control genetically modified neurons that express light-sensitive ion channels, such as channelrhodopsin-2 [27–29]. In this approach, a specific population of neurons are genetically encoded or targeted with viral vectors to express light-activated ion channels, allowing them to depolarize in response to illumination. This can trigger a cascade of depolarization that propagates across the cortex. This method provides exceptional spatial and temporal control, enabling researchers to precisely regulate the onset, location, and threshold of SD, which is difficult to achieve with traditional techniques like electrical or chemical stimulation. The precision of optogenetics, allowing cell-type-specific, non-invasive, and reproducible induction of SD justifies the need for genetic modification via transgenic or viral-vector-mediated expression.

The ability to adapt this method to make it minimally or non-invasive combined with its high precision, makes it an ideal and reproducible method for studying the mechanisms and dynamics of SD in real-time. Non-invasive optogenetic SD induction has shown to reproduce migraine-like behaviors (photophobia, allodynia) and hemodynamic/metabolic signatures seen in migraine patients, strengthening the translational impact [1, 25].

Minimally invasive optogenetic induction approaches have provided key insights into inflammatory pathways activated by SD [30], and have been shown to mimic key features of spontaneous SDs, both electrographically (SD threshold and propagation rate) and behaviorally (SD-evoked behaviors like full body stretching) in migraine with aura mice [31]. A recent advancement enables the optogenetic induction of SD via a minimally invasive, wireless system in freely behaving mice. This platform allows for repeated SD induction and provides a powerful tool to investigate the long-term consequences of SDs in mouse models of neurological pathologies, including stroke and migraine with aura [32].

## Electrophysiological detection of SD

Electrophysiology remains the gold standard for the detection and characterization of SDs. Conventionally, researchers employ DC-coupled electrocorticography (ECoG) arrays to capture the hallmark negative shift in direct current potential that defines an SD. However, traditional metal-based electrodes, while widely used for neural recordings, are poorly suited for detecting DC-coupled signals. Their limited low-frequency response and susceptibility to electrode polarization introduce significant distortions, obscuring the true dynamics of slow phenomena such as SDs [33]. In contrast, solution-filled silver wire glass micropipettes offer superior DC recording performance due to their high input impedance and minimal polarization, enabling more faithful capture of SD dynamics. Nonetheless, their invasive nature and restriction to a few localized recording sites limit their ability to resolve the large-scale spatiotemporal patterns of SD propagation.

Recent advances in materials science, such as graphene solution-gated field-effect transistors (gFETs), provide a promising alternative. These devices exhibit high sensitivity, flexibility, and scalability, enabling real-time monitoring of the slow, large-amplitude voltage shifts characteristic of SDs with both high spatial resolution and multisite recording capability [29, 34]. Traditionally, reliable detection of SD has required invasive placement of electrodes directly on or within the brain, such as via intracortical probes, ECoG arrays, or epidural electrodes in small animals. This necessity arises because the skull severely attenuates, distorts, and filters the slow, localized electrical signals produced during SD. A major technical goal is to achieve high-fidelity, through-skull electrophysiological recordings of SD.

**Fig. 1:**
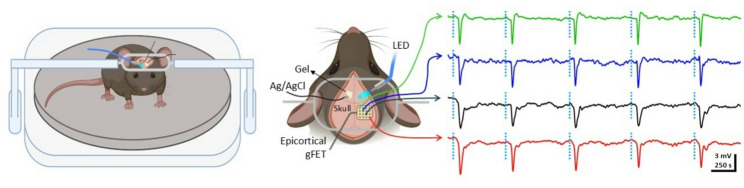
Setup used for non-invasive induction and electrographic detection of SDs in awake mice. Electrophysiological recordings in head-fixed mice involve surgery for attaching the headplate. Several days later, mice are habituated to the head-fixation apparatus. Commonly a treadmill or a running ball is used in lab-built equipment or in commercially available systems such as Neurotar, a floating floor gives the animal the sensation that is moving despite head-fixation. A graphene micro-transistor array (gFET) is placed subdermally, as is a silver pellet for reference on the contralateral hemisphere. A blue LED is used for optogenetic induction of SDs through the intact skull on demand (dashed vertical blue lines). Using this approach we have shown ( [29] and unpublished data in this figure) that SDs are reliably induced and, although there is some signal attenuation, SDs of between 5–10 mV are clearly detected electrographically through the mouse skull. This preparation is also compatible with simultaneous neuroimaging techniques such as laser speckle contrast imaging, intrinsic optical signal or functional ultrasound imaging. Created with BioRender.com

We recently demonstrated that minimally invasive, subdermal recordings using graphene micro-transistor arrays enable reliable detection of SDs [29]. Although some loss of spatiotemporal detail and waveform fidelity occurs due to volume conduction through the skull, subdermal recordings clearly captured depolarization waves (5–10 mV) propagating at approximately 3 mm/min, along with associated neuronal activity suppression. Combining through-skull graphene transistor recordings with non-invasive optogenetic induction of cortical SDs in head-fixed mice offers a powerful new platform to investigate SD dynamics in awake mice ( [29]; Fig. 1).

SD is usually recorded at the brain surface, but multichannel depth probe studies challenge the assumed equivalence between SD and spreading depression. They show that SD induces variable effects, from suppression to amplification of activity across cortical layers depending on its depth of penetration. Therefore, to gain a full picture of SD dynamics on neuronal activity through cortical columns depth probes are required [35].

With the development of electrodes fabricated from novel materials exhibiting favorable impedance and stimulation characteristics [36], impedance-based methods [37] may offer new strategies for detecting SDs without directly stimulating neuronal activity. During SD, extracellular space contraction and ionic gradient collapse produce characteristic shifts in local tissue impedance. Importantly, impedance monitoring can be integrated with concurrent electrophysiological recordings, providing a complementary approach for studying SD without introducing additional artifacts. Future advances may further enhance the utility of these techniques.

**Fig. 2:**
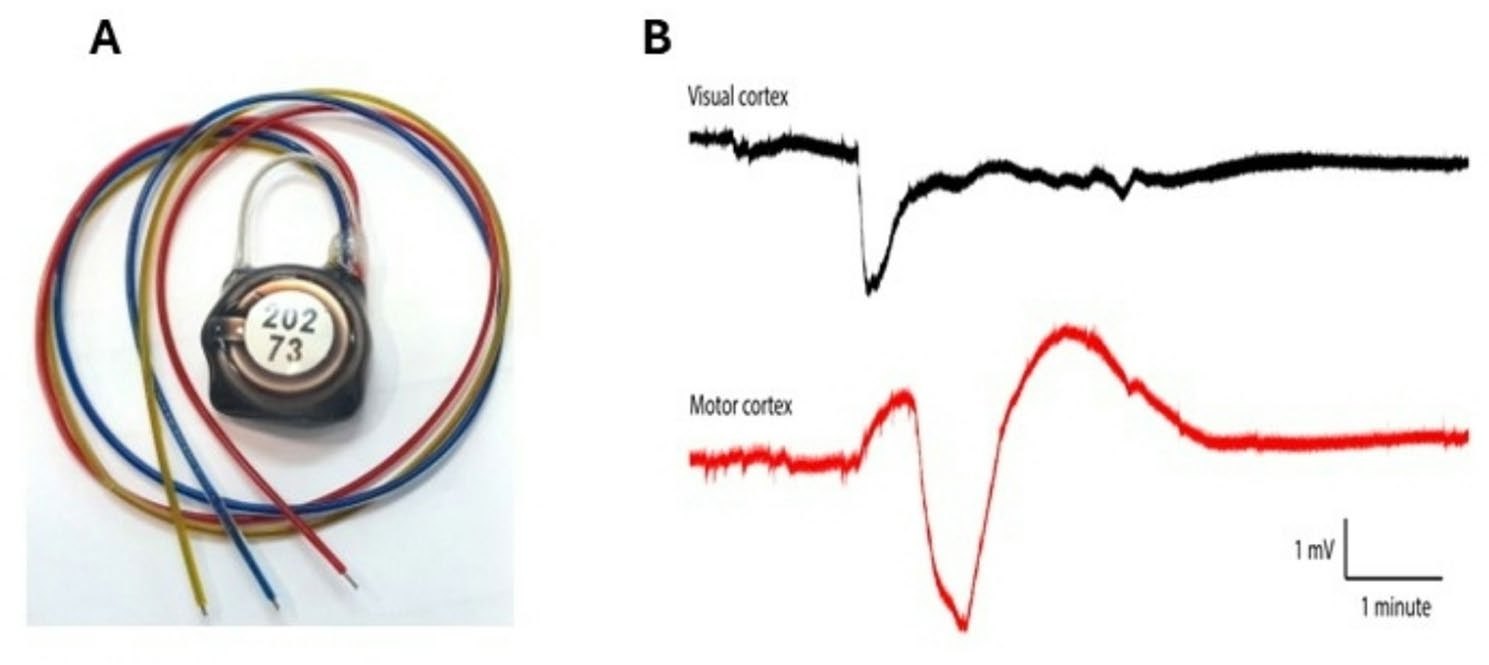
DC-coupled wireless telemetry devices allow detection of spontaneous SD events in rodent models of neurological disorders. **A** Commercially available DC-coupled wireless telemetry device (Open Source Instruments) **B** Example of a spontaneous SD detected using 2 leads, one in the visual cortex and the other in ipsilateral motor cortex in a mouse model of migraine with aura (authors unpublished raw data)

Continuous long-term electrographic recordings are essential to study spontaneous SD events in disease-relevant models. While tethered systems enable several days of recording, improving animal welfare and extending recording durations to weeks or months requires wireless, DC-coupled telemetry. Ongoing efforts are addressing this need, exemplified by the Open Source Instruments DC-coupled telemetry device, which enables continuous recordings exceeding one month in freely moving rodents (see Fig. 2).

## Methods used to study blood flow responses to SD

Accurate detection of SDs requires direct electrographic recordings to capture the characteristic negative DC shift; however, a variety of imaging modalities have been developed to visualize the associated blood flow responses, enabling indirect detection of SD initiation and the tracking of its spatiotemporal propagation across the cortex.

The intense neuroglial depolarization during SDs trigger significant and dynamic cerebral hemodynamic responses. In both humans and rodents, in normoxic brain, a slight initial hypoperfusion is sometimes observed [3, 38]. This is followed by a substantial increase in regional cerebral blood flow (CBF; i.e., hyperemia), which usually lasts up to 3 min, and aims to meet the increase energy demand and clear the extracellular space from released cortical metabolites [3, 15]. Often the hyperemia gives way to a prolonged post-SD oligemia that reduces CBF (10–40%) below baseline for an hour or longer before normalizing [1, 39]. However, it is noteworthy that differences between mice and rats have been observed. For example, SD-evoked hemodynamic responses are more pronounced in mice, with a 60–70% initial hypoperfusion observed, followed by a decline in CBF of 60% for up to an hour [1, 40].

The hemodynamic changes evoked by SDs have been characterized in experimental and/or clinical studies with a variety of methodologies at a macroscopic/mesoscopic level. These include observing the associated blood flow changes with laser Doppler or laser speckle contrast imaging (LSCI), recording diffusion/perfusion changes with magnetic resonance imaging (MRI), and/or measuring reflectance changes using intrinsic optical signal (IOS) imaging [41–44]. As shown in Table 2, these methods of studying SDs have specific advantages and disadvantages (reviewed in [45, 46]). In brief, available non-invasive techniques have sufficient spatiotemporal resolution to observe the initial hypoperfusion that coincides with the DC shift, the peak hyperemia and the sustained post-SD oligemia [39, 47]. Nevertheless, given the limited measurement depth of these techniques at the microvascular level (with the exception of MRI), the hemodynamic responses during SDs are mostly derived based on the observation of pial vessels and cortical arterioles close to the surface of the brain [48, 49]. As different segments and vascular compartments might respond differently to SDs [48], it is evident that novel techniques with higher spatiotemporal resolution, in particular depth, are needed to fully comprehend SD-evoked hemodynamic responses. Functional brain imaging modalities such as functional MRI (fMRI) and positron emission tomography have excellent brain penetration; however they are not frequently performed on awake rodents due to challenges associated with head-motion during scanning and the need for restrain training for anesthesia-free imaging [50, 51]. Alternatively, functional ultrasound (fUS) imaging is a method that has acquired popularity in preclinical research studies as it allows imaging brain-wide activity in awake head-fixed or tethered mice in a minimally-invasive way (discussed below) [38, 52].

## Mesoscopic level imaging to study SDs

Mesoscopic imaging techniques encompass the intermediate scale (100 μm-5 mm) between macroscopic (large-scale brain networks, > 5 mm) and microscopic (single neurons, 0.1–100 μm) spatial domains [53, 54], offering valuable insights into neuronal network properties and their dysfunction in disorders such as epilepsy and migraine [55, 56].

Optical imaging techniques, particularly IOS imaging and LSCI, have emerged as powerful tools to visualize SDs in awake rodents. IOS leverages changes in light reflectance associated with alterations in blood volume and oxygenation during SD events, offering high spatial resolution over wide cortical areas. LSCI, on the other hand, measures dynamic changes in blood flow and can detect the vascular signature of SDs in real time (Table 2). They provide a powerful alternative over traditional macroscopic methods for investigating SDs [54, 57]. Importantly these methods are compatible with awake head-fixed approaches.

**Table 2:** Comparison of commonly used SD detection techniques in rodents

Technique	Principle	Spatial resolution (µm)	Temporal resolution (ms)	Signal/Target	Advantages	Limitations	Typical use case	Invasiveness
Electrophysiology (ECoG, DC-coupled)	Directly measures neuronal activity (extracellular field potentials)	~ 10–250	~ 0.1-1	Electrical potential shifts	High temporal resolution (ms), direct SD detection, established technique	Movement artifacts, baseline drift	Tracking SD onset/propagation dynamics	Yes
Impedance Monitoring	Impedance increases with cell swelling/edema	~ 200–300	~ 1–10	Tissue conductivity/resistance changes	Passive detection, minimal perturbation, can integrate with other methods	Less spatial detail, less used method, needs stable baseline	Supplementary tracking of SD waves	Yes
Intrinsic Optical Signal Imaging (IOS)	Measures backscattered light changes from hemoglobin/cellular swelling	~ 50–100	~ 10–100	Changes in cortical reflectance (blood volume/oxygenation)	High spatial resolution, wide-field view, label-free	Limited depth, slower signals (~ seconds), sensitive to motion	Mapping SD propagation across cortex	No
Laser Speckle Contrast Imaging (LSCI)	Detect changes in laser speckle patterns caused by moving red blood cells to measure surface blood flow	~ 10–100	~ 10–100	Blood flow dynamics	Fast imaging of hemodynamic response, real-time mapping	Cannot measure direct neural activity, surface-limited	Vascular effects of SDs	No, but cranial windows (adults) improve quality and depth of image
Fluorescent imaging of ions/neurotransmitters i.e. Widefield Calcium Imaging (GCaMP versions)	Uses genetically encoded indicators. Fluorescence intensity increases with calcium concentration	~ 50–100	~ 33–100	Neuronal calcium influx (activity proxy)	Indirect visualization of cellular depolarization, genetically targeted	Requires transgenic lines or viral expression, photobleaching	Neuronal recruitment during SDs	Yes
Magnetic Resonance Imaging	Leverages magnetic properties to generate spatially resolved maps of tissue structure, metabolism and hemodynamics	~ 100,000-500,000 (0.1–0.5 mm)	~ 1000-10,000	Multiparametric (blood flow dynamics, blood oxygenation, apparent diffusion coefficient)	Whole brain 3D imaging of hemodynamic responses, tracks multiple physiological correlates of SDs (edema, metabolic changes)	Motion sensitivity (requires immobilization); cannot measure direct neural activity	Multiparametric effects of SDs cortical and subcortical structures	No
Functional Ultrasound Imaging (fUS)	Quantifies cerebral blood volume	~ 100	~ 100–1000	Blood flow dynamics	Whole brain fast imaging of hemodynamic response, real-time mapping	Cannot measure direct neural activity	Vascular effects of SDs cortical and subcortical structures	Cranial windows (adults)
Microscopic level imaging (2-photon/miniscopes)	Uses non-linear excitation enabling deeper tissue penetration	~ 1–5	~ 30-1000	Blood flow dynamics and biosensors	Tissue or cellular fast imaging of hemodynamic response, real-time mapping	Cannot capture large-scale SD dynamics	Vascular effects of SDs cortical and subcortical structures	Yes

All techniques are available in awake rodents

Fluorescent mesoscopic imaging has emerged as a powerful tool to study SD in the brain, particularly with the advent of genetically encoded indicators [54, 57, 58]. By using fluorescent indicators that report changes in intracellular calcium, transmembrane voltage, or key signaling molecules like potassium, glutamate, acetylcholine, and serotonin, researchers can monitor the complex dynamics of SD across large cortical areas [57, 59–62]. Additionally, multi-color mesoscopic imaging facilitates the study of defined cellular populations, enhancing our understanding of the cellular and molecular processes underlying SD. Compared to point electrophysiology, mesoscopic imaging has the ability to analyze both the amplitude and spatiotemporal trajectories of individual SDs across the cortex [60].

**Fig. 3:**
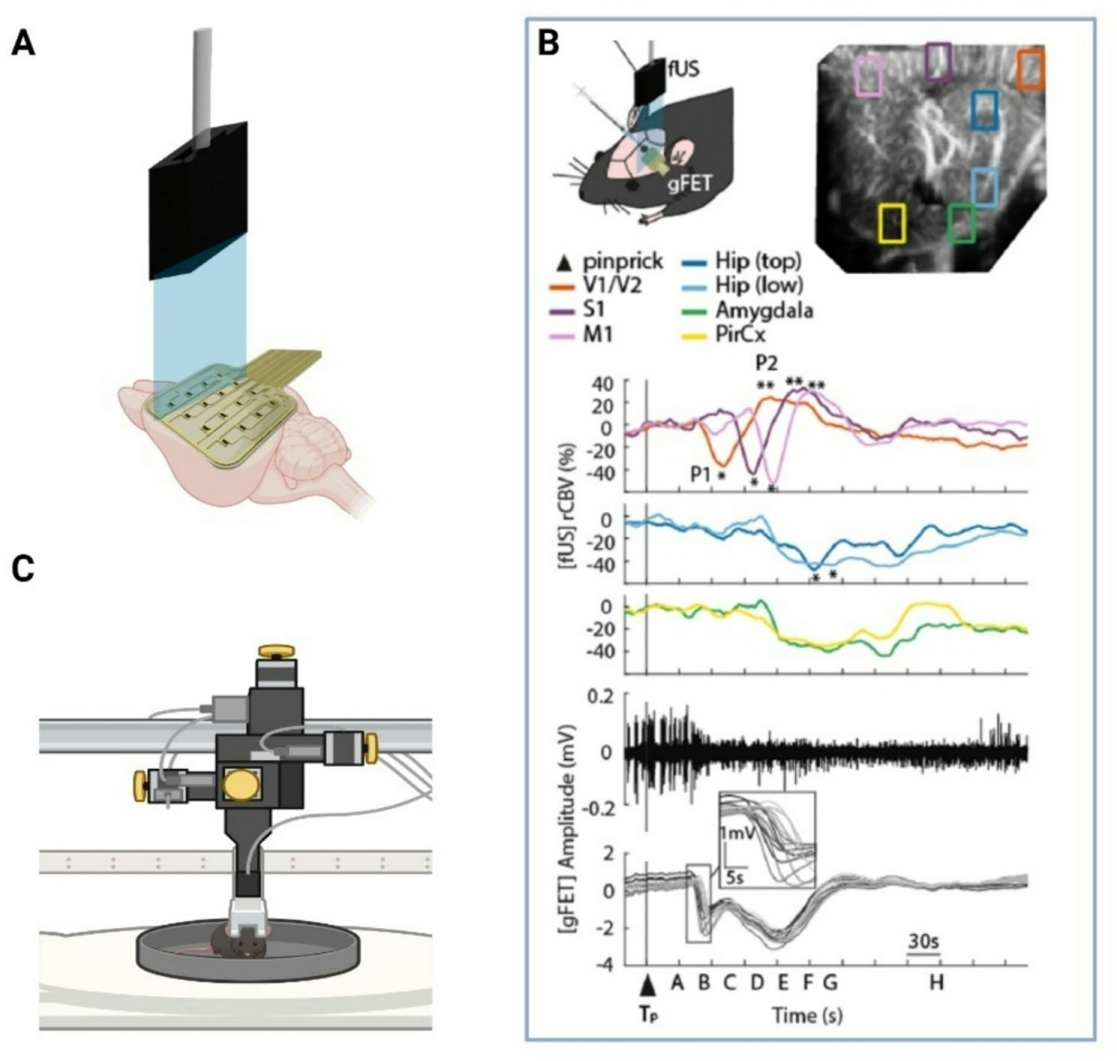
Advanced technological platform that allows detection of SD using a DC-coupled graphene micro-transistor array (gFET), concurrently with functional ultrasound imaging (fUS) of cerebral blood volume. **A** fUS permits quantification of SD-evoked hemodynamic changes in both superficial and subcortical areas at the mesoscopic level and is compatible with simultaneous DC-coupled electrophysiology. **B** Upper panel shows the experimental protocol and multiple rectangles indicating different extracted regions of interest. The middle panel shows the time course of cerebral blood volume changes in visual (V1/V2, orange) areas, somatosensory cortex (S1, brown), motor cortex (M1, pink), hippocampus (blue), amygdala (green) and piriform cortex (yellow). Lower panel shows the AC-coupled electrographic response recorded from a single transistor (top, black trace), and DC-coupled detection of SD from multiple transistors, with blow up illustrating propagation (below, grey traces). Reproduced from Ref [38]. with permission from the Royal Society of Chemistry (Creative Commons Attribution 4.0 license). **C** This approach is also compatible with head-fixed or tethered awake mice. Created with BioRender.com

FUS imaging is an emerging minimally-invasive technique that can track, with high spatiotemporal resolution, dynamic changes during brain activation [63]. It is an ultrasensitive and quantitative technique that can monitor in real time cerebral blood volume and flow (including neurovascular coupling), providing highly sensitive anatomical and functional mapping of the vasculature [48]. Moreover, fUS imaging also allows the study of a variety of subcortical brain regions such as the thalamus and hypothalamus [64], which are relevant in migraine pathophysiology and have been shown to be affected by SDs in FHM mouse models [65, 66], providing the ability to monitor superficial and deep brain structures concurrently. As shown in Fig. 3, an additional advantage of fUS imaging is that it is compatible with electrophysiology [38] and awake setups, permitting visualization of brain dynamics through the intact skull of young mice or via cranial windows in older animals, making it suitable for investigating SD-evoked network and behavioral effects in awake rodents.

FUS imaging also enables dynamic and non-invasive measurements of brain-wide vascular activity at a microscopic scale during task-evoked activities in rodents [63]. This is possible due to the intravenous injection of microbubbles that allows ultra-high resolution and can distinguish between arterioles and veins, providing local estimates of vascular dynamics such as CBF and speed [63], which are likely to be affected by SDs. In this regard, SD-induced hemodynamic changes, including cerebral blood volume and CBF velocity during SD, have been recorded in single penetrating arterioles or venules of the cerebral cortex of anaesthetized wild-type rodents [48]. fUS can be compatible with awake setups and has been used to investigate SDs following focal cortical ischemia in awake rats [52]. As FUS imaging cannot readily assess precapillary sphincters and pericytes at first-order capillaries, which limits the monitoring of capillary blood flow [67], supplementary techniques are needed to study SD-evoked changes at a microscopic level.

## Microscopic level imaging to study SDs

**Fig. 4:**
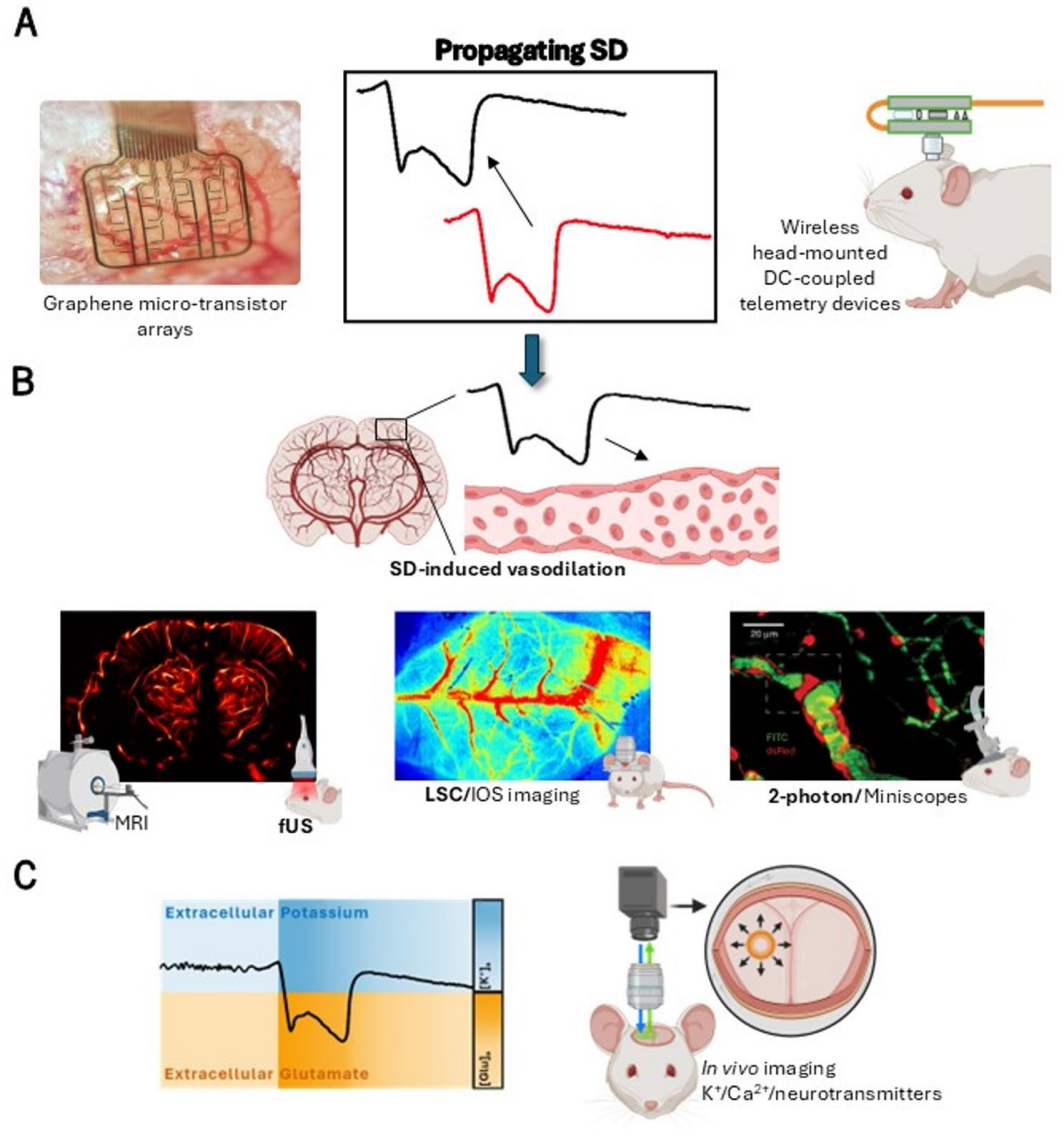
Diagram mapping current state-of-the-art techniques for the detection of spreading depolarizations (SDs) and their associated downstream effects. **A**. The gold standard for detection of SDs is electrophysiology (middle panel). Cutting-edge approaches include multisite graphene micro-transistor arrays for acute recordings (left panel; authors unpublished data) and DC-coupled wireless telemetry for long-term evaluation of spontaneously occurring SDs (right panel). **B**. A common consequence of SDs, in particular in migraine with aura, is an increase in cerebral blood flow (middle upper panel) which can be detected with over a number of spatial scales. Macroscopic level techniques include magnetic resonance imaging and functional ultrasound (fUS) permitting monitoring global vascular responses (left panel; authors unpublished data). Mesoscopic level techniques include laser speckle contrast (LSC) and intrinsic optical signal (IOS) imaging permitting monitoring blood flow changes over large cortical areas (middle lower panel; reproduced from [44] licensed under a Creative Commons Attribution 4.0 license). Microscopic level techniques include two photon microscopy and miniscopes that offer high spatial resolution allowing imaging of individual capillaries (right panel; reproduced from [70] under a Creative Commons Attribution 4.0 license). **C**. SDs evoke large changes in ionic concentrations and neurotransmitters levels, for example increases in extracellular potassium and glutamate (left panel). These can be monitored using either fluorescent dyes or more commonly genetically encoded indicators. Tracking fluorescent signatures allows visualization of SDs initiation and propagation across the cortex (right panel). Large changes in ionic concentrations together with cell swelling can also be detected by impedance measurements (not shown). Created with BioRender.com

Advanced microscopy techniques allow to visualize the impacts of SDs at a tissue or (sub)cellular level, due to both high-resolution and super-resolution of these imaging techniques (Fig. 4). At a cellular level, SDs trigger an increase influx of ions and water causing swelling of neurons, distortion of dendritic spines (dendritic bedding) and shrinkage of the extracellular space, a process known as cytotoxic edema [3, 68]. Two-photon laser scanning microscopy is a technique that enables real-time visualization of SD-evoked cytotoxic edema in neuronal and glial cells [69]. In this regard, it has been shown that in addition to morphological changes, SDs induce a transient intracellular acidification and mitochondrial depolarization only in neurons, suggesting that astrocytes are not the primary contributor to SD propagation [69].

Two-photon microscopy also allows imaging through a cortical column, and as such, it has been used to study the mechanisms that precede SD initiation, SD propagation as well as SD-induced hemodynamic changes in different cortical layers [70]. Parker et al., revealed that spontaneous and frequent neuronal plumes of glutamate release initiated SDs in awake FHM 2 mice [71]. Grubb et al., showed in anaesthetized mice that precapillary sphincters of penetrating arterioles maintain and control capillary flow, whereas SDs constricted persistently the sphincters and caused vascular trapping of blood cells [70]. These effects are believed to contribute to the prolonged post-SD oligemia and may be involved in the increased risk of stroke observed in patients with migraine with aura [9, 70].

*In vivo* two-photon (2P) microscopy allows taking time-lapse three-dimensional images stacks through thinned-skull of fluorescently tagged transgenic mice, before, during and after SDs [72]. In this respect, it was recently shown in anaesthetized mice that two population of immune cells residing in the meninges respond differently after SDs, with pial macrophages responding instantly and dural macrophages responding after 20 min [72]. However, it is still unclear whether activation of these immune cells modulates head pain. As it is possible to conduct 2P or 3P imaging in awake head-fixed or tethered mice [73], microscopic investigations of SDs is a possibility in awake brain.

Miniaturized fluorescence microscopes (miniscopes) can be used to monitor vascular dynamics and neuronal activity at cellular resolution in awake rodents [74, 75]. Compared to optical imaging methods which are generally performed by fixing the heads of rodents under the microscope objectives; miniscopes can be carried by rodents, enabling real-time monitoring of vascular dynamics and neuronal activity in tethered animals (reviewed in [76]). To look at the superficial cortex and deeper brain structures, it is possible to implant miniscopes into other migraine-relevant structures such as the cranial meninges, and trigeminal ganglion. Rasmussen et al., showed that solutes in CSF released by SDs, such as CGRP, directly activated trigeminal ganglion neurons in anaesthetized mice [15]. Degel et al. monitored meningeal vascular dynamics in awake mice before and after administration of levcromakalim, a vasodilator known to induce migraine in humans [75]. By providing real-time visualization of neurovascular coupling at a microscopic level, these techniques can offer unique insights into migraine pathophysiology (Table 2). While head-fixation enables performing awake neuroimaging methods at the macroscopic, mesososcopic and microscopic spatial scales (Fig. 4), there are technical challenges and ethical considerations associated with this approach. Minor movement artifacts can degrade data fidelity requiring motion-correction algorithms for microscopic recordings, whereas fixation imposes stress through restraint, demanding habituation protocols prior to experimentation, duration limits (ideally less than 1–2 h per session) and welfare monitoring to minimize distress. Awake neuroimaging protocols are needed and should be regularly reviewed and updated to assess complex migraine phenomena such as photophobia or SD propagation while refining the welfare of the animals. Despite these limitations, we believe this approach enhances the scientific value of the experiments relative to those conducted under anesthesia.

## Future

Electrophysiology remains the only method capable of directly detecting SDs, making it the current gold standard. All other approaches infer SD indirectly by observing downstream effects (Fig. 4). However, voltage imaging can provide a rapid readout of membrane potential changes and is amenable to mesoscopic imaging approaches allowing tracking of voltage signals over large cortical areas [77] and can be amenable to awake recordings [78]. Future advances in genetically encoded voltage-sensitive indicators may enable direct optical detection of SD. If these indicators become sensitive enough for through-skull detection, and if the required imaging and stimulation hardware can be made sufficiently lightweight, a fully wireless, all-optical system for both optogenetic induction and voltage-sensitive detection of SD in freely moving rodents could become feasible. Such a system would open the door to chronic, non-invasive monitoring over extended periods, without restricting natural behavior, providing unique insights into migraine complex pathophysiology.

## Conclusion

Novel neuroimaging techniques have revolutionized our ability to monitor SD and its impacts, spanning spatial scales from dendrites to the whole brain. Moreover, the combination of these imaging techniques with in vivo electrophysiology in awake rodents, allows a deeper understanding of how SDs affect neuronal circuits in real-time without the confounding effects of anesthesia. We believe that implementation of these methods in awake brain will close the translational gap and improve the relevance of preclinical animal models of migraine with aura.

## Data Availability

No datasets were generated or analysed during the current study.

## Abbreviations

CBF: Cerebral blood flow
fUS: Functional ultrasound
IOS: Intrinsic optical signal
LSCI: laser speckle contrast imaging
MRI: Magnetic resonance imaging
SD: Spreading depolarization
